# Young and Aged Neuronal Tissue Dynamics With a Simplified Neuronal Patch Cellular Automata Model

**DOI:** 10.3389/fninf.2021.763560

**Published:** 2022-01-07

**Authors:** Reinier Xander A. Ramos, Jacqueline C. Dominguez, Johnrob Y. Bantang

**Affiliations:** ^1^Instrumentation Physics Laboratory, National Institute of Physics, College of Science, University of the Philippines, Quezon City, Philippines; ^2^Institute for Neurosciences, St Luke's Medical Center, Quezon City, Philippines; ^3^Elderly and Dementia Care, Institute for Dementia Care Asia, Quezon City, Philippines; ^4^Computational Science Research Center, University of the Philippines, Quezon City, Philippines

**Keywords:** neuronal dynamics, continuous cellular automata, brain, numerical model, activation function, aged neurons

## Abstract

Realistic single-cell neuronal dynamics are typically obtained by solving models that involve solving a set of differential equations similar to the Hodgkin-Huxley (HH) system. However, realistic simulations of neuronal tissue dynamics —especially at the organ level, the brain— can become intractable due to an explosion in the number of equations to be solved simultaneously. Consequently, such efforts of modeling tissue- or organ-level systems require a lot of computational time and the need for large computational resources. Here, we propose to utilize a cellular automata (CA) model as an efficient way of modeling a large number of neurons reducing both the computational time and memory requirement. First, a first-order approximation of the response function of each HH neuron is obtained and used as the response-curve automaton rule. We then considered a system where an external input is in a few cells. We utilize a Moore neighborhood (both totalistic and outer-totalistic rules) for the CA system used. The resulting steady-state dynamics of a two-dimensional (2D) neuronal patch of size 1, 024 × 1, 024 cells can be classified into three classes: (1) Class 0–inactive, (2) Class 1–spiking, and (3) Class 2–oscillatory. We also present results for different quasi-3D configurations starting from the 2D lattice and show that this classification is robust. The numerical modeling approach can find applications in the analysis of neuronal dynamics in mesoscopic scales in the brain (patch or regional). The method is applied to compare the dynamical properties of the young and aged population of neurons. The resulting dynamics of the aged population shows higher average steady-state activity 〈*a*(*t* → ∞)〉 than the younger population. The average steady-state activity 〈*a*(*t* → ∞)〉 is significantly simplified when the aged population is subjected to external input. The result conforms to the empirical data with aged neurons exhibiting higher firing rates as well as the presence of firing activity for aged neurons stimulated with lower external current.

## 1. Introduction

Since the development of the first neuronal model by Louis Lapicque in 1907, most neuronal models we have today use a set of ordinary differential equations (ODEs) to model the dynamics of neurons (Lapicque, 1907; Brunel and Van Rossum, 2007). The Nobel-prize winning Hodgkin-Huxley (HH) model describes the relationship between the membrane potential of the neuron and the flow of ions across the membrane normally via the ion channels (Hodgkin and Huxley, 1952; Gerstner et al., 2014). The HH model is successfully used to describe the dynamics of a squid giant axon and even the Purkinje fibers in the heart (Noble, 1962). Other models such as Dalton and FitzHugh (1960); Nagumo et al. (1962) and Morris and Lecar (1981) models were improvisations and simplifications on the HH model. While these models are good representations of a neuronal response, it is a challenge for us to construct a simple model useful in describing the behavior of a large neuronal population. HH neurons can be arbitrarily interconnected (Pang and Bantang, 2015) but simulations for large numbers of neurons take long computational run time and need high computing resources since they require solving many coupled ODEs and saving numerous system variables.

One study involves cortical simulations of 10^9^ neurons of a cat using Blue Gene/P supercomputer (Ananthanarayanan et al., 2009). The simulations were powered by 147, 456 CPUs and 144 TB of main memory (roughly ~6 × 10^3^ neurons/CPU, ~144 KB/neuron). In this study, we propose simple cellular automata models to simulate many interconnected neurons that will help investigate integrated dynamics of up to millions (10^6^) of neurons using lower CPU and GPU requirements. Our simulations are powered with 1 CPU and 16 GB of memory (RAM) (roughly ~10^6^ neuron/CPU, ~16 KB/neuron). The Blue Brain project primarily uses the NEURON simulation environment to accomplish their feat. NEURON mainly solves ODE-based models with data-driven parameters. However, solving ODEs differs from the cellular automata (CA)-based models. CA models can employ a look-up-table-based algorithm that is usually faster than solving ODEs.

Cellular automaton modeling paradigm was first developed in the late 1940's by Stanislaw Ulam and John von Neumann (von Neumann, 1966). It became popular after it was used to model Conway's Game of Life in the 1970's. A CA system A consists of the set C of agents or “cells” *c* (c∈C) arranged in a lattice L with a specified neighborhood set $N=C^{n+1}$ where *n* is the number of neighbors of any given cell. Certain boundary conditions are also applied depending on the properties of the physical system being modeled (Wolfram, 2002; Arciaga et al., 2009). These cells have assigned state *s*, typically obtained from a finite state binary set such S={0,1}, being the simplest. The “0” and “1” states usually represent either “dead” or “alive,” or for our present case of neuronal dynamics represent “resting” or “spiking” (active), respectively. Each neighborhood has a unique state $\bar{s}\in S^{n+1}$.

The various dynamics of a CA model also emerge from the rules applied to the lattice. In this work, we investigate a CA system with a first-order linear approximation to the HH neuronal response as our rule for each cell. The activation function is further discussed in section 2.1. We perform different analyses (spatiotemporal, cobweb, bifurcation) on the CA system to classify the observed dynamics. This lays the groundwork of our proposed model that can be extended to future directions. In section 7, we extended our model into a nonlinear activation function, which is used to better fit the response of young and aged neurons of a rhesus monkey (Coskren et al., 2015).

Aged neurons have distinctly less myelin and shorter axon internodal distance leading to reduced conduction velocity (Peters, 2007). Dysregulated signaling pathways in oligodendroglia and the loss of reregenerative capacity of oligodendrocyte progenitor cells are thought to be the major cause for myelin loss (Rivera et al., 2021). At the synapse, dendritic spines where majority of excitatory synaptic processes occur are smaller and lesser (Pannese, 2011) but are functionally intact to make synaptic connections. The connections may be weaker but exhibit lesser capacity for short-term plasticity (Mostany et al., 2013). Presumably, the shrinkage in the density of the dendritic spine impacts excitatory synaptic activity in neuronal circuits and accounts for the cognitive changes observed in older adults even in the absence of pathology. However, in the light of reports of increase in action potential firing rates (excitability) in aged neurons, there is need for studies to further understand the dynamics of cell-to-cell communication and open avenues for potential interventions to mitigate the effects of brain aging. In a study on rhesus monkey prefrontal cortex (Coskren et al., 2015), it was found that aged neurons typically have higher action potential (AP) firing rates compared to younger neurons. The empirical data from the study is used as an application of our CA model.

## 2. Continuous Cellular Automata Model of a Neuronal Patch

As a CA model, neurons are arranged in a two-dimensional lattice L composed of 1, 024 × 1, 024 cells. This choice of lattice size is one of the highest possible in a common computing device (without the need of high-performance computing). The resulting dynamics does not change with varying lattice size (Ramos and Bantang, 2018, 2019c). However, the computing performance is compared in section 9. The state of *s* each neuron is represented by a real number *a* (stands for activity) which ranges from 0 to 1, thus forming a continuous-state CA. The state of each neuron is initialized by assigning a random value to the CA state drawn from a uniform distribution such that *a*_*i, j*_∈[0, 1] for all CA cells in the system (*i, j*∈[1, 1024]).

**Figure 1:**
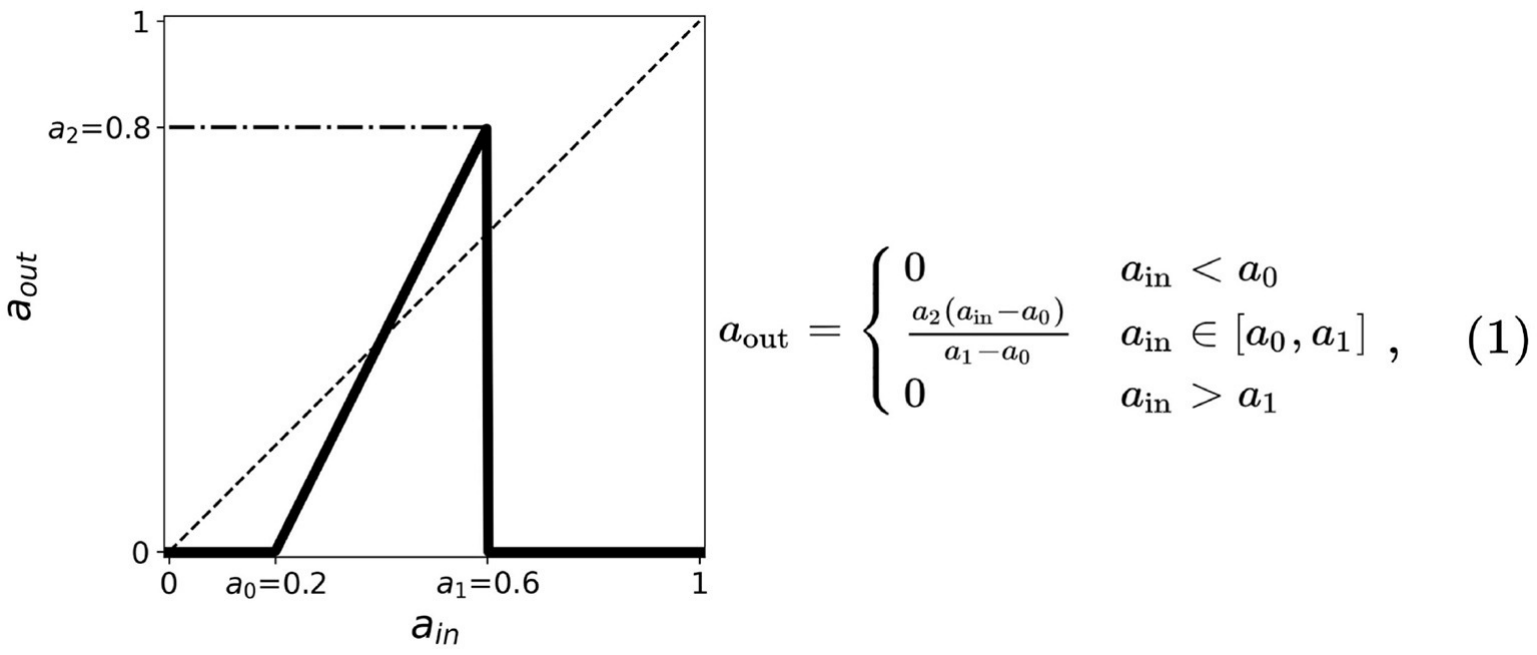
A simplified neuronal response used for the CA model of neurons. The output *a*_out_ is related to the probability of the neuron to fire given the input *a*_in_. The plot shows such functions for threshold values *a*_0_ = 0.2, *a*_1_ = 0.6, *a*_2_ = 0.8. The dashed line corresponds to *a*_out_ = *a*_in_ useful in the cobweb analysis.

At each timestep, the average state of the neighborhood of a given cell is taken as the cell's input *a*_in_ or stimulus. The response of the current cell is obtained from the mapping of *a*_in_ into its corresponding output *a*_out_ or response. A generalized linear activation or response function is shown in Figure 1. The various modes of neighborhood and boundary conditions are discussed in section 2.2 and the activation function is discussed in section 2.1 below. We found that a value of 100 timesteps is enough to achieve steady-state for any initial state, and that randomizing the initial location of active cells does not affect the dynamical results of our model (Ramos and Bantang, 2018; Ramos, 2019).

### 2.1. Activation Function

The activation function used in the CA model is mainly derived from the response function of the HH model. Many other neuronal models such as leaky integrate-and-fire (Tal and Schwartz, 1997; Gerstner et al., 2014) and Wilson-Cowan (Wilson and Cowan, 1972) exhibit a similar trend of neuronal firing rate with increasing input current. Three main properties of the activation function can be observed:

1. Two thresholds (a minimum and a maximum) in the input are present indicating that neurons fire only when stimulated by an input current between these two thresholds. We, respectively, assign for these the thresholds *a*_0_ and *a*_1_, the minimum and maximum.
2. The firing rate monotonically increases whenever the input current is between *a*_0_ and *a*_1_; the firing rate is zero otherwise.
3. A maximum threshold in the output is present limiting the firing rate values for the entire range of *a*_in_. We assign this as *a*_2_.

The thresholds are incorporated into the activation function and are simplified by taking the first-order approximation as described in Figure 1. The parameter thresholds are varied from 0 to 1 with a step size of 0.1. The condition *a*_0_ = *a*_1_ results in a trivial mapping *a*_out_ = 0, for all *a*_in_-values. The resulting equation for the neuronal activation function is given by:


(1) $$a_{\text{out}}=\;\{\begin{array}{ll} 0 & a_{\text{in}}<a_{0}\\ \frac{a_{2}(a_{\text{in}}-a_{0})}{a_{1}-a_{0}} & a_{\text{in}}\in [a_{0},a_{1}]\\ 0 & a_{\text{in}}>a_{1} \end{array}$$

We performed an exhaustive search by varying each parameter in {*a*_0_, *a*_1_, *a*_2_} from 0 to 1 with increments of 0.1 (Ramos and Bantang, 2018, 2019c). The resulting steady-state dynamics for all possible combinations of {*a*_0_, *a*_1_, *a*_2_} in this scheme were classified into one of the types discussed in section 3.

### 2.2. Neighborhood and Boundary Conditions

**Figure 2:**
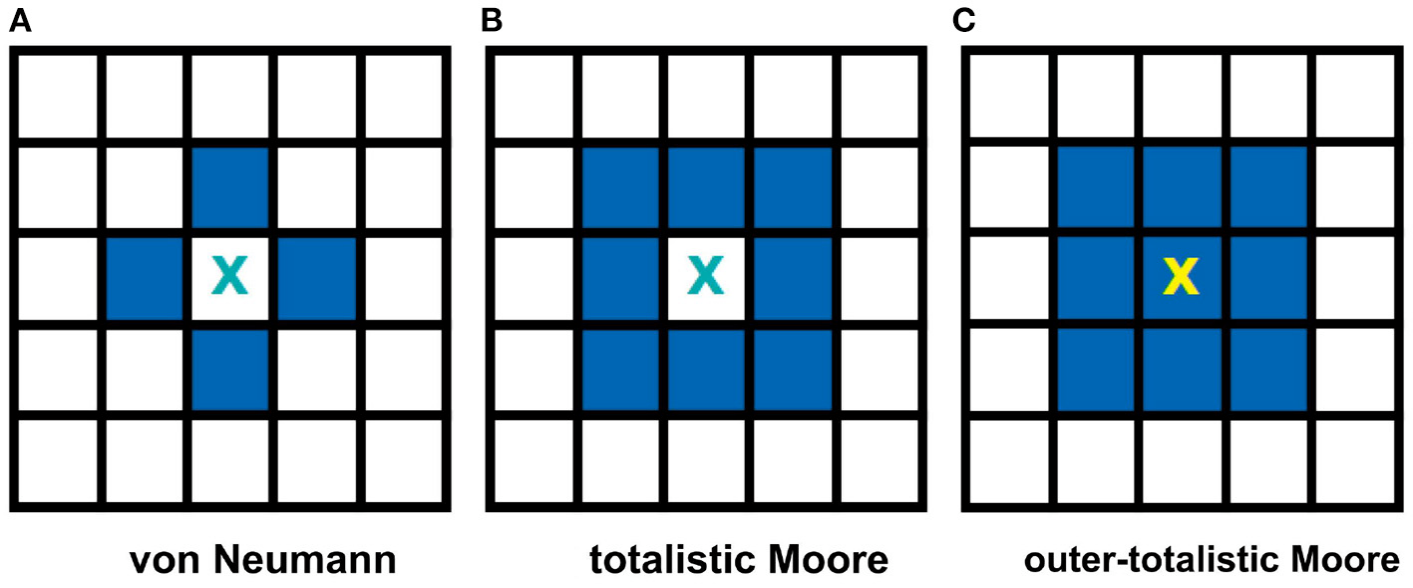
Common neighborhood configurations used in CA theory. The shaded cells show the neighbors of cell X for each type of neighborhood. The von Neumann neighborhood **(A)** of cell X consists of the four cells in the primary directions, while Moore **(B)** extends it to the secondary directions. In an outer-totalistic setting **(C)**, the cell X itself is included in the neighborhood. In this work, Moore neighborhood is used because a biological neuron is typically connected to all neighboring cells in the 2D space.

Two often used neighborhood configurations in CA models are the von Neumann and the Moore neighborhoods (Wolfram, 2002). Figure 2A, on one hand, shows a von Neumann setting. In this case, the central cell of any 3 × 3 subset of the lattice is connected to the adjacent cells in the primary directions (4 neighbors: left, right, top, and bottom) with respect to the cell. Figure 2B, on the other hand, shows a Moore neighborhood setting. This time, the central cell is connected to the adjacent cells in the primary and secondary directions (including the diagonal directions, total of 8 neighbors). Moore neighborhood is used in the model since a biological neuron is typically connected to all neighboring cells in the 2D space (Hawick and Scogings, 2011). Two types of Moore neighborhood configurations are considered: totalistic and outer-totalistic. The only difference between these configurations is that the outer-totalistic setting has the central cell of the 3 × 3 subset included in the neighborhood state (see Figure 2C).

**Figure 3:**
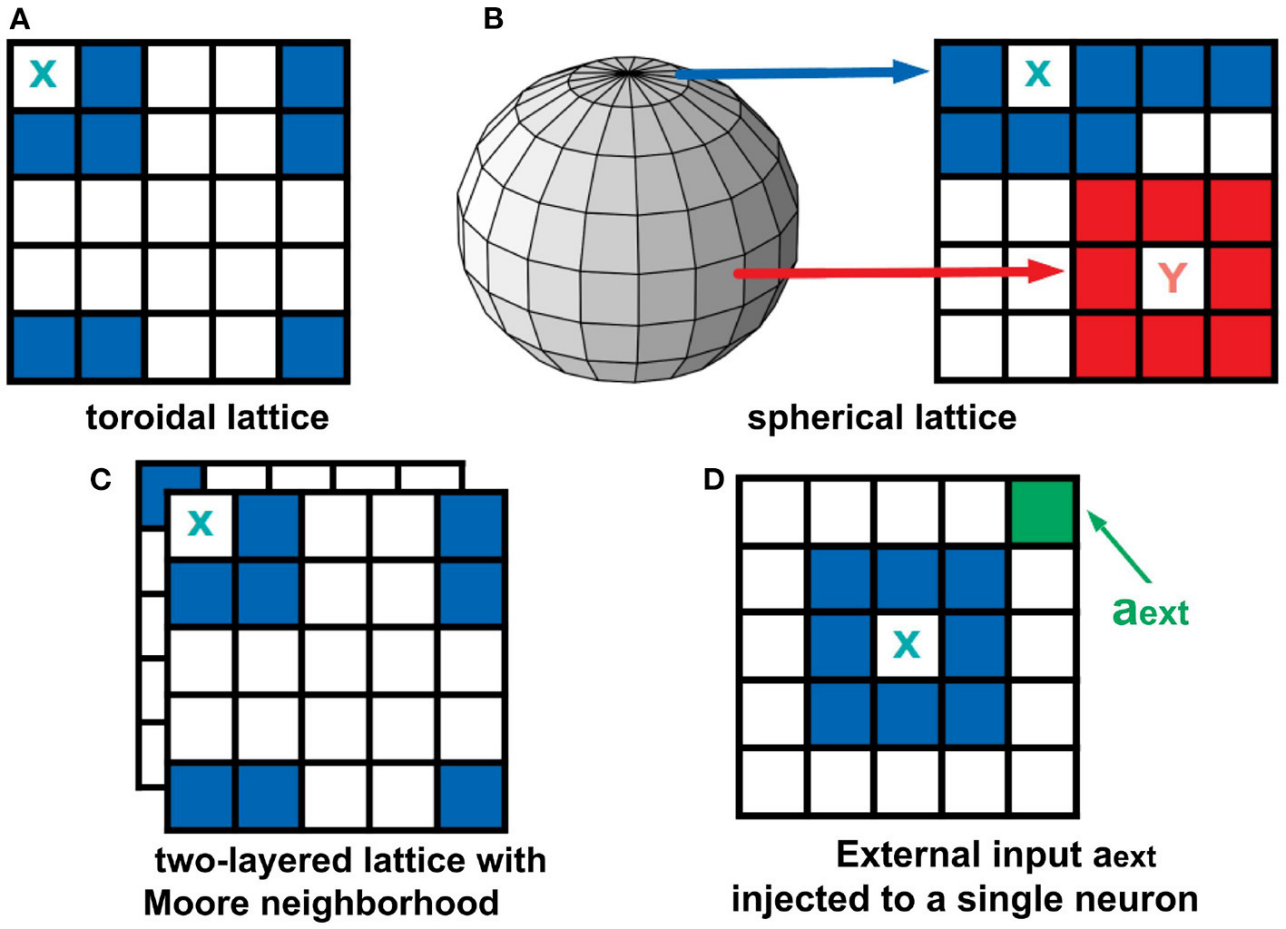
Top row: Boundary conditions used in this study. In a toroidal lattice **(A)**, we wrap-around the top and bottom rows, and the leftmost and rightmost parts of the grid. For the spherical lattice **(B)**, a Mercator projection was used to draw the lattice on the sphere's surface. Bottom row: Extended neighborhood and boundary conditions explored in this work. For the two-layered lattice **(C)**, the intra-layer connection is Moore, but the inter-layer connection is the overlay between layers. For the analysis of external input **(D)**, a fraction of neurons in the population are set to be always active *a*_ext_ = 1.

The boundary conditions describe how the cells at the edge of the lattice behave. Two types of boundary conditions were considered: toroidal and spherical boundaries. With the toroidal boundary condition, the cells on the leftmost column are connected to the rightmost column, and the top row is connected to the bottom row. This produces a wrap-around effect on our automaton as shown in Figure 3A. For the spherical setting (Ramos and Bantang, 2019c), the square lattice is projected on the surface of a sphere (Mercator projection) as shown in Figure 3B. Observe that the cells in the top (and bottom) row are fully connected to each other becoming a pole, while in the middle rows neighbors wrap around.

### 2.3. Two-Layered Lattice and the External Input

To analyze a quasi three-dimensional (3D) neighborhood, we extended our analysis to a two-layered automaton for both toroidal and spherical boundary conditions (Ramos and Bantang, 2019a,b). The intra-layer connection has a Moore neighborhood setting, while the inter-layer connection is a direct overlay between the layers. The neighborhood conditions for this two-layer lattice is visualized in Figure 3C. For systems with the number of layers greater than two, the topmost and bottommost layer are connected as if the bottommost layer is stacked above the topmost layer.

A CA system (Ramos and Bantang, 2019a) with a constant external input *a*_in_ = *a*_ext_ = 1 injected to one of the neurons *c*_ext_, shown in Figure 3D, is also analyzed. In this case, the neuron *c*_ext_ is always in spiking state since *a* = 1 at all times.

## 3. Numerical Experiments

**Figure 4:**
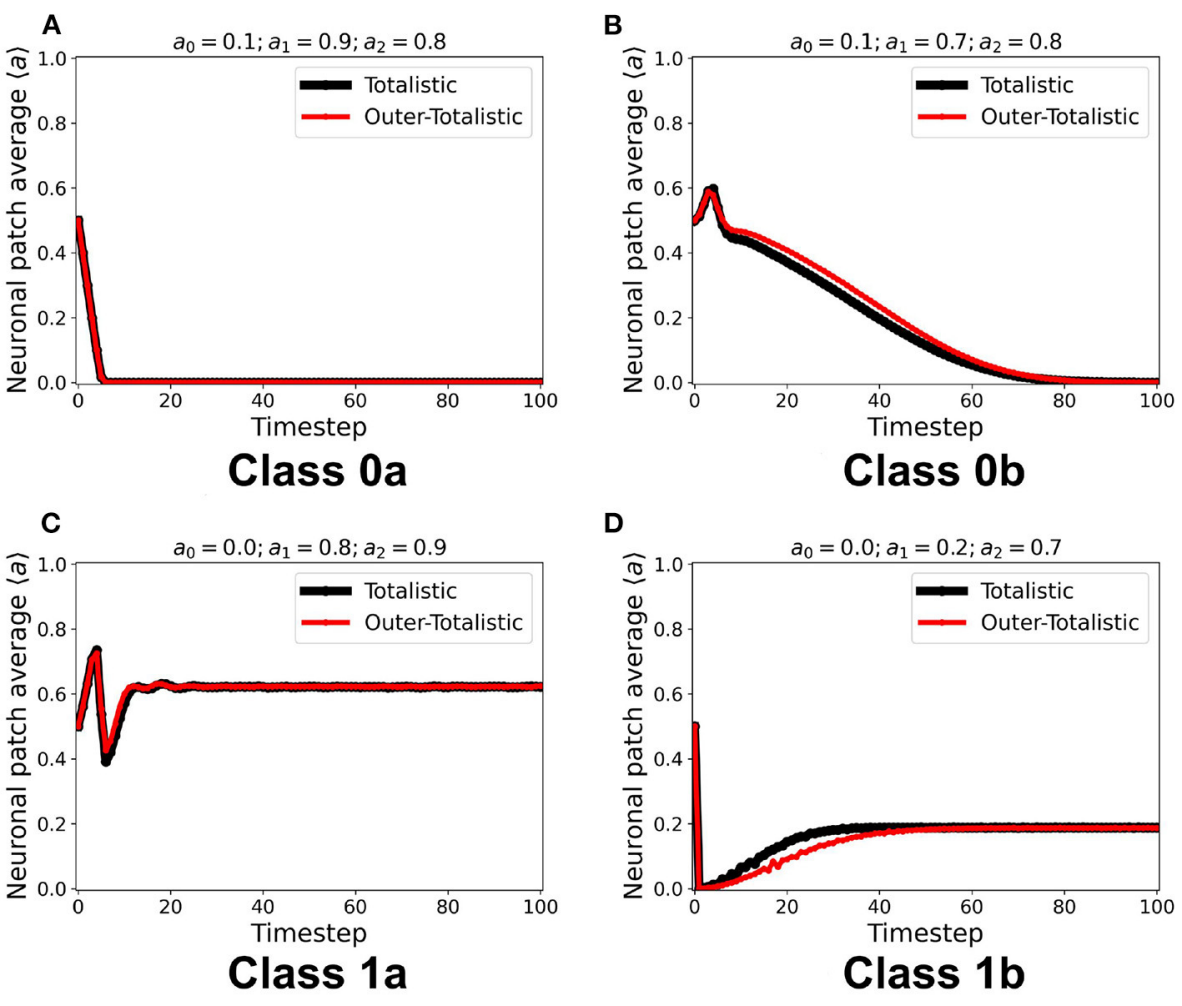
**(A–D)** Representative steady-state dynamics for each neuronal CA class. The steady-state is taken as the average neuronal activity of the patch at that timestep. The black solid line shows the average neuronal activity using totalistic rules while the red solid line corresponds to the outer-totalistic setting.

**Figure 5:**
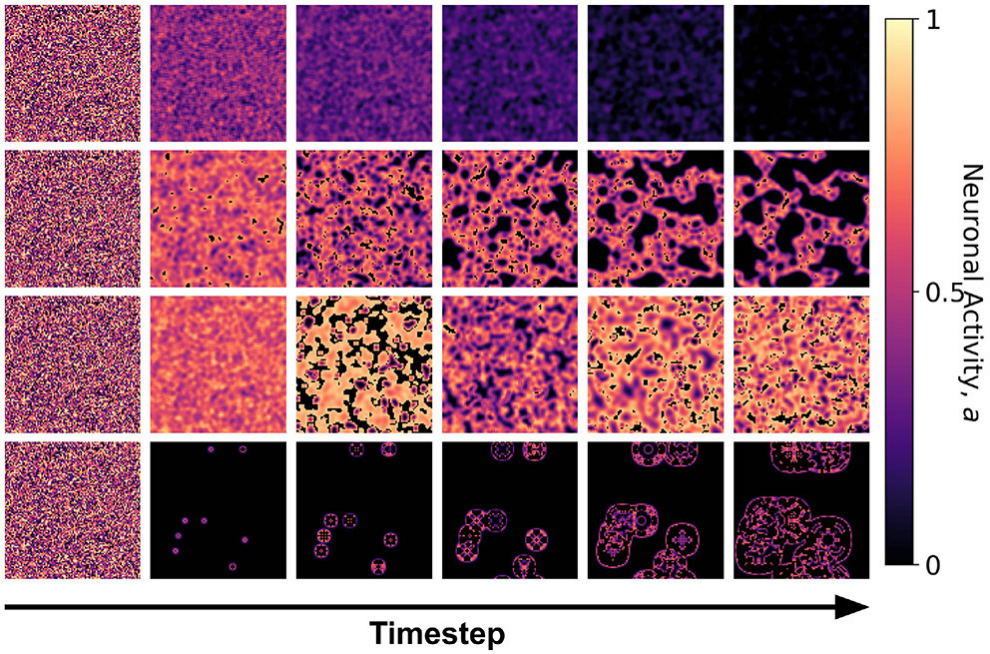
Snapshots of the neuronal CA with toroidal lattice configuration for each class taken at different timesteps of the simulation. The snapshots are taken with increasing timesteps (not necessarily consecutive) to highlight the different dynamics observed. At *t* = 0, the initial state is the same for all classes, but each class evolved into one of the CA classes, depending on the set of parameter thresholds used in the activation function.

We first examined the dynamics of the neuronal CA using Moore toroidal boundary condition. The average neuronal patch activity 〈*a*〉 is obtained for each timestep and plotted as shown in Figure 4. We observed two types of steady-state dynamics: a quiescent or zero steady-state; and a spiking or nonzero steady-state. These steady-state trends are also observed in the HH model (as well as Morris-Lecar) as Type I and Type II neurons, respectively (Hodgkin and Huxley, 1952; Morris and Lecar, 1981; Gerstner et al., 2014). The steady-state dynamics is the same for totalistic and outer-totalistic neighborhoods. Samples of spatiotemporal activity of the neuronal CA are shown in Figures 5, **7**, respectively for toroidal and spherical shapes. We observed that a certain subset of the spiking steady-state CA produced exploding patterns before reaching a randomly spiking steady-state. For any given parameter set *a*_0_, *a*_1_, *a*_2_, the dynamics are observed to fall into any one of the following classes.

1. Class 0: Quiescent Steady-State: (a) Fast-decay; (b) Slow-decay.
2. Class 1: Spiking Steady-State:(a) With random patterns; (b) With exploding patterns.

**Figure 6:**
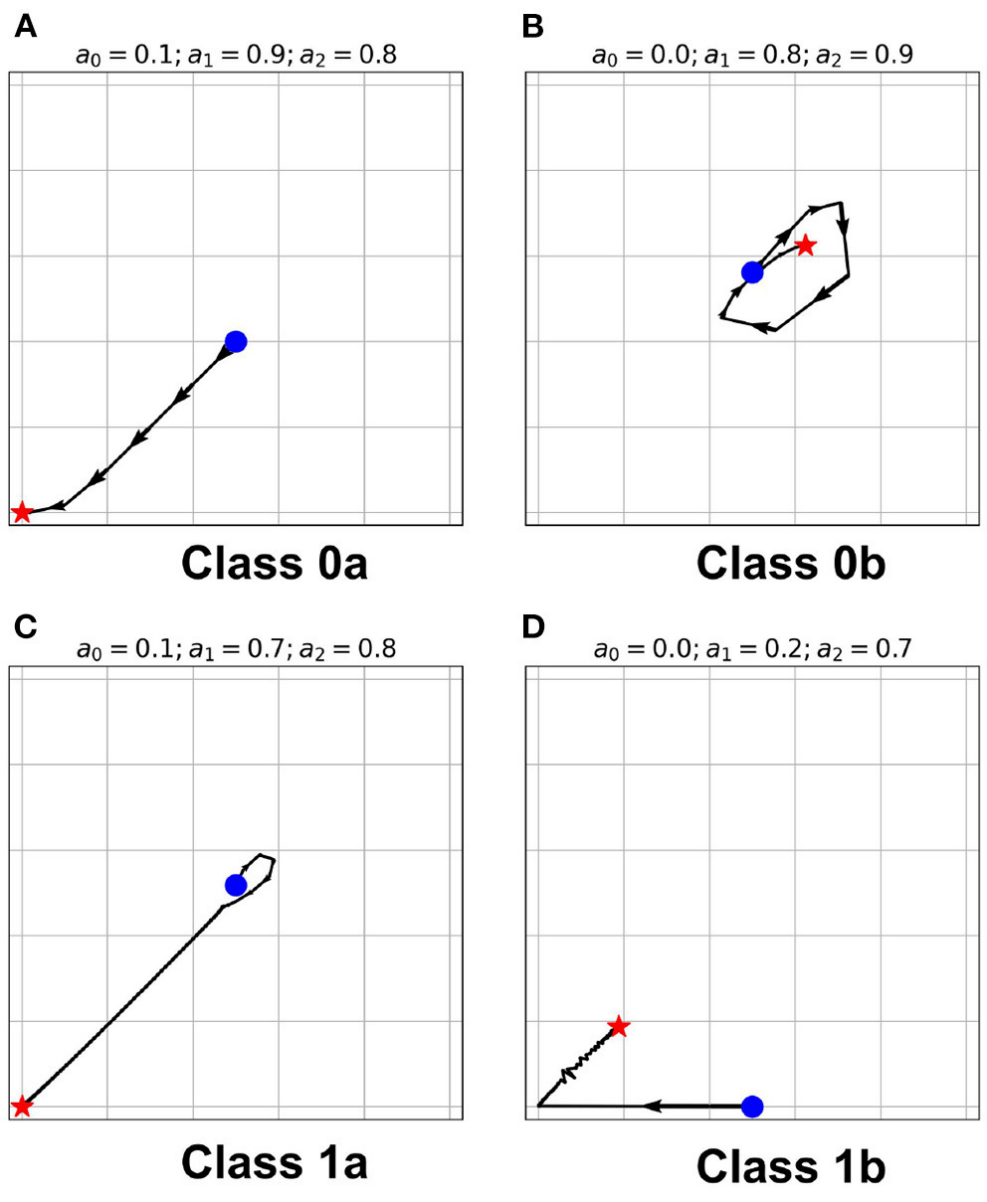
**(A–D)** State-space trajectories of each of the classes. The 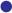 and 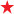 marks the automaton state at *t* = 0 and *t* = ∞, respectively. The arrows show the direction of evolution of the average neuronal steady-state over time.

**Figure 7:**
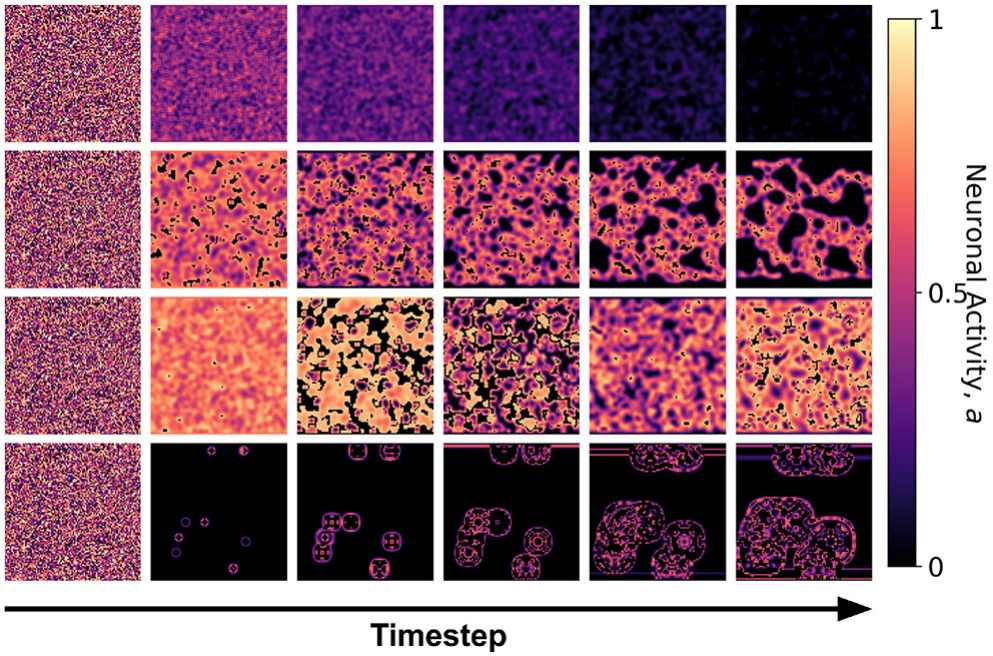
Snapshots of the neuronal CA with spherical lattice configuration for each class taken at different timesteps of the simulation. The snapshots are taken with increasing timesteps (not necessarily consecutive) to highlight the different dynamics observed. At *t* = 0, the initial state is the same for all classes, but each class evolved into one of the CA classes, depending on the set of the parameter threshold used in the activation function.

The classification above becomes more obvious as we look at the steady-state trajectory shown in Figure 6. Here, we plotted the activity *a*_*t*+1_ vs. *a*_*t*_. With spherical boundary conditions, this steady-state dynamics remains unchanged (see Supplementary Figure 1). Hence, the boundary condition in the systems investigated does not affect neuronal CA classification. There is a slight variation on the spatiotemporal evolution of the automaton with the spherical boundary condition as shown in Figure 7. The effect of spherical lattice is clearly visible on Class 1b, where the activity signal bounces back from the location of the polar-points (top and bottom rows).

In a previous work (Ramos and Bantang, 2019c), we explored the different regimes in which these CA classes exist in the phase space diagram. We found that a minimum of 20% of the population of the neurons must be active or spiking at *t* = 0 to observe a nonzero steady-state CA (Class 1). As the output threshold *a*_2_ is increased, the systems with parameters that fall near the phase boundary, transitions from Class 0 to Class 1 CA, and thus, increasing the region for which Class 1 CA is observed. In this work, the chosen set of parameters belong to the stable regions in which the dynamical classification is observed.

## 4. Effect of External Input and Layered Lattice

**Figure 8:**
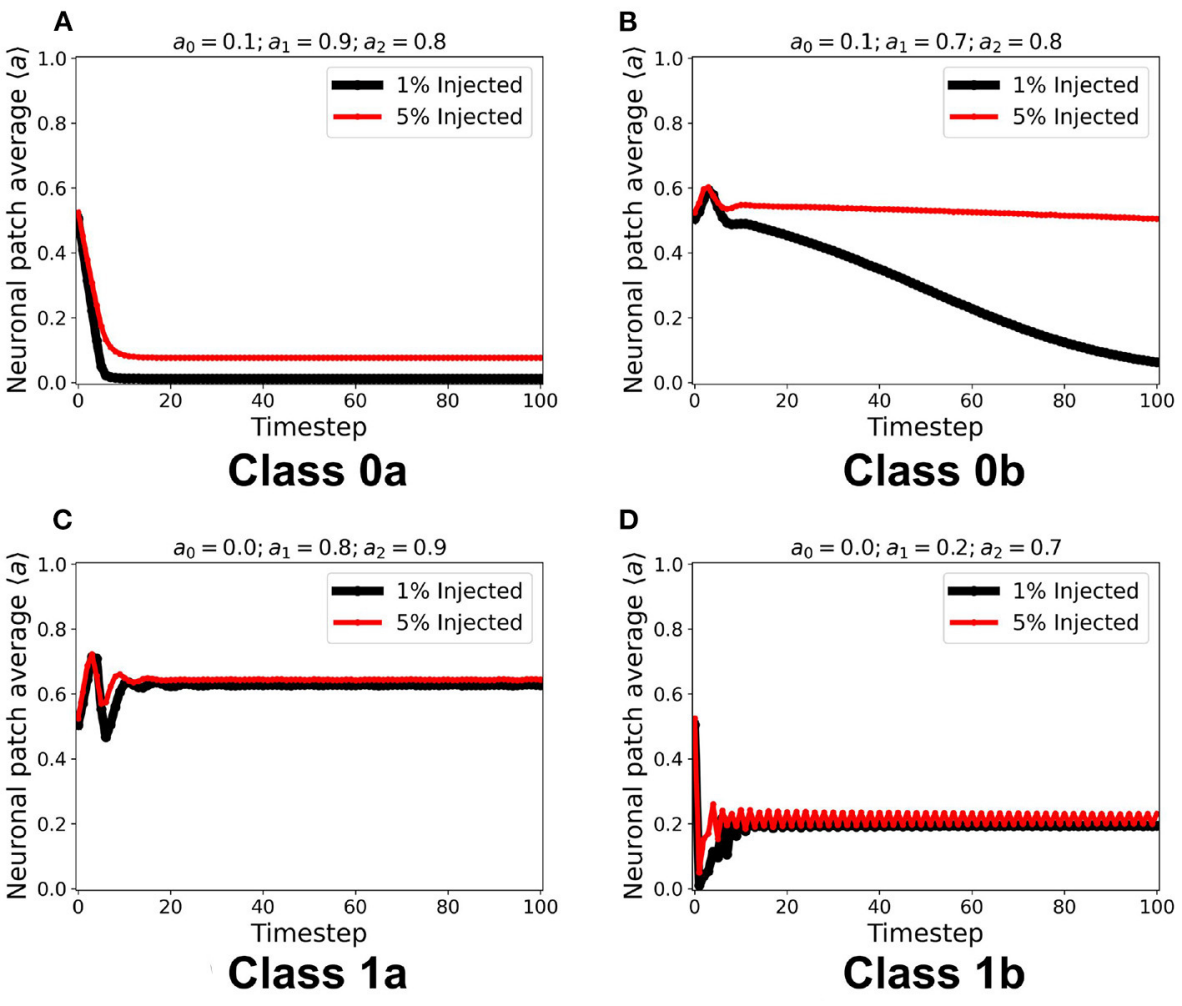
**(A–D)** Steady-state dynamics for each CA class in a toroidal lattice configuration with external input injected to a fraction of the neuronal population. The black line shows the average steady-state when 1% of the population is injected with external constant input. The red line corresponds to 5% of the neuronal population injected accordingly.

Using the same initial state of the automaton, we assigned a certain fraction (1% and 5%) of the neurons in random locations to be *c*_ext_, injected with constant *a*_in_ = 1. It is notable in Figure 8 that for all steady-state classes, the overall system activity 〈*a*〉 becomes typically much greater than the input. Class0b neurons with 5% *c*_ext_ were found to have similar steady-state dynamics with Class 1a. Furthermore, Class 1b neurons resorted to an oscillating steady-state with 5% *c*_ext_.

**Figure 9:**
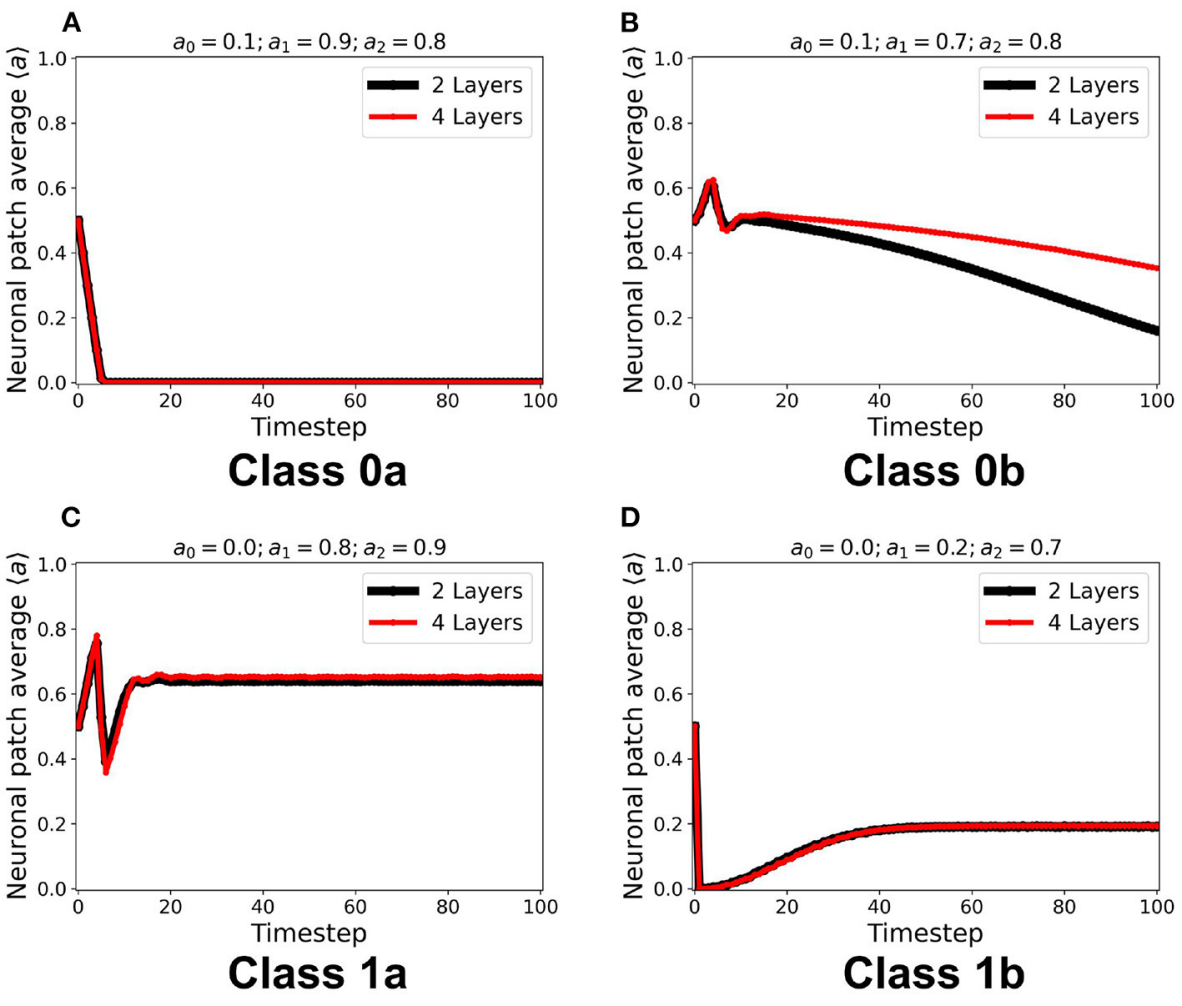
**(A–D)** Steady-state dynamics of each class in a two-layered (black line) and four-layered (red line) toroidal lattice configuration. The dynamical trend remains the same for each class.

We implemented the method described in section 2.3 for a two-layered and four-layered CA system. The average steady-state activity remains unchanged across each layer and remains the same for the whole CA system (see Supplementary Figure 2). Increasing the number of layers from two to four layers did not change the CA steady-state classification. It is notable in Figure 9 that increasing the number of layers also increases the number of neurons in the neighborhood state, and consequently delays the transition to quiescent steady-state in Class 0b. The delay is also due to the gradual decrease of the wave amplitude as it travels at least once across the system. This follows the proportionality between the system size and the time taken for the signal to propagate across the system (Wolfram, 2002).

## 5. Cobweb Diagram Analysis

Cobweb diagrams visualize how a dynamical system behaves over time (Stoop and Steeb, 2006). Consider a CA system response function defined by *a*_out_ = *f*(*a*_in_) (see Figure 1). We then can draw a cobweb diagram on a plane (*x, y*) = (*a*_in_, *a*_out_) as follows:

1. Given a chosen starting point (*x*_start_, *y*_start_) = (*a*_start_, 0), we trace a vertical line from it to (*a*_start_, *f*(*a*_start_)).
2. We trace a horizontal line from (*a*_start_, *f*(*a*_start_)) until it crosses the dashed line with the equation *a*_out_ = *a*_in_. This value becomes the new starting point, such that (*x*_start_, *y*_start_) = (*f*(*a*_start_), *f*(*a*_start_)).
3. Repeat steps 1 and 2 until we reach a sufficient number of steps (here, we use 100).

**Figure 10:**
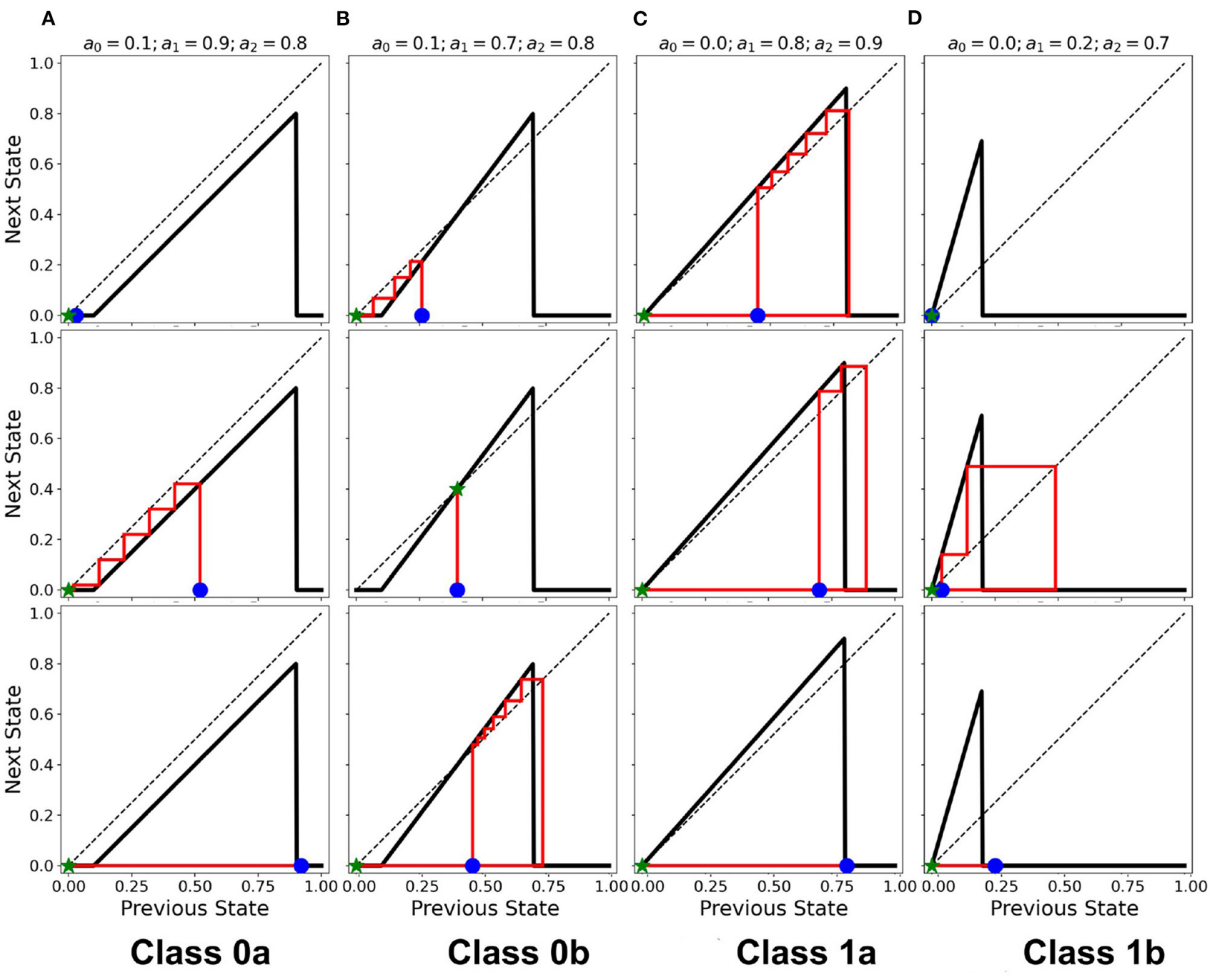
**(A–D)** Cobweb diagrams showing the behavior of individual neurons for each class. In each column, the individual plots show different cases that a neuron is initialized (marked by 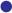). Each case may not necessarily be the same across all the classes. The ⋆ marks the final state on the x-axis with the red line showing the trajectory of the neuronal state through time.

The resulting cobweb diagrams for the different dynamical classes are shown in Figure 10 (Ramos and Bantang, 2020). The activation function of Class 0a falls below the line *a*_out_ = *a*_in_ such that any neuron transitions to quiescent state regardless of its initial state (marked by the blue star ⋆). A collection of neurons of this class approaches a quiescent state in a short amount of timesteps. However, in Class 0b, the activation function crosses the line *a*_out_ = *a*_in_ once. Any neuron state that starts from the left or right of the intersection point results in a temporary overall active state but the system eventually ends up to be in the quiescent steady-state. If the neuron state starts exactly at the intersection point, its state remains there as a trivial application of the procedure above. Only a few of neurons coincide with this trivial case since the initial state-values of the CA in our numerical experiments is obtained from a uniform random distribution in the range [0, 1]. Collectively, Class 0b neurons go into the quiescent state but at a slower rate compared to Class 0a. Inhibitory neurons (Type I) can therefore be modeled by Class 0 neuronal patch.

If the intersection point is located at the origin (i.e. *a*_0_ = 0), the collection of neurons always approaches a spiking steady-state. The greater is the difference *a*_1_−*a*_0_, the higher the average steady-state value 〈*a*〉 of the system. Neuronal CAs with lower average steady-state value usually result from exploding patterns (Class 1b). Random patterns (Class 1a) consequently produce higher average steady-state values. Excitatory (Type II) neurons belong to Class 1 in the classification scheme presented.

## 6. Bifurcation Diagram Analysis

**Figure 11:**
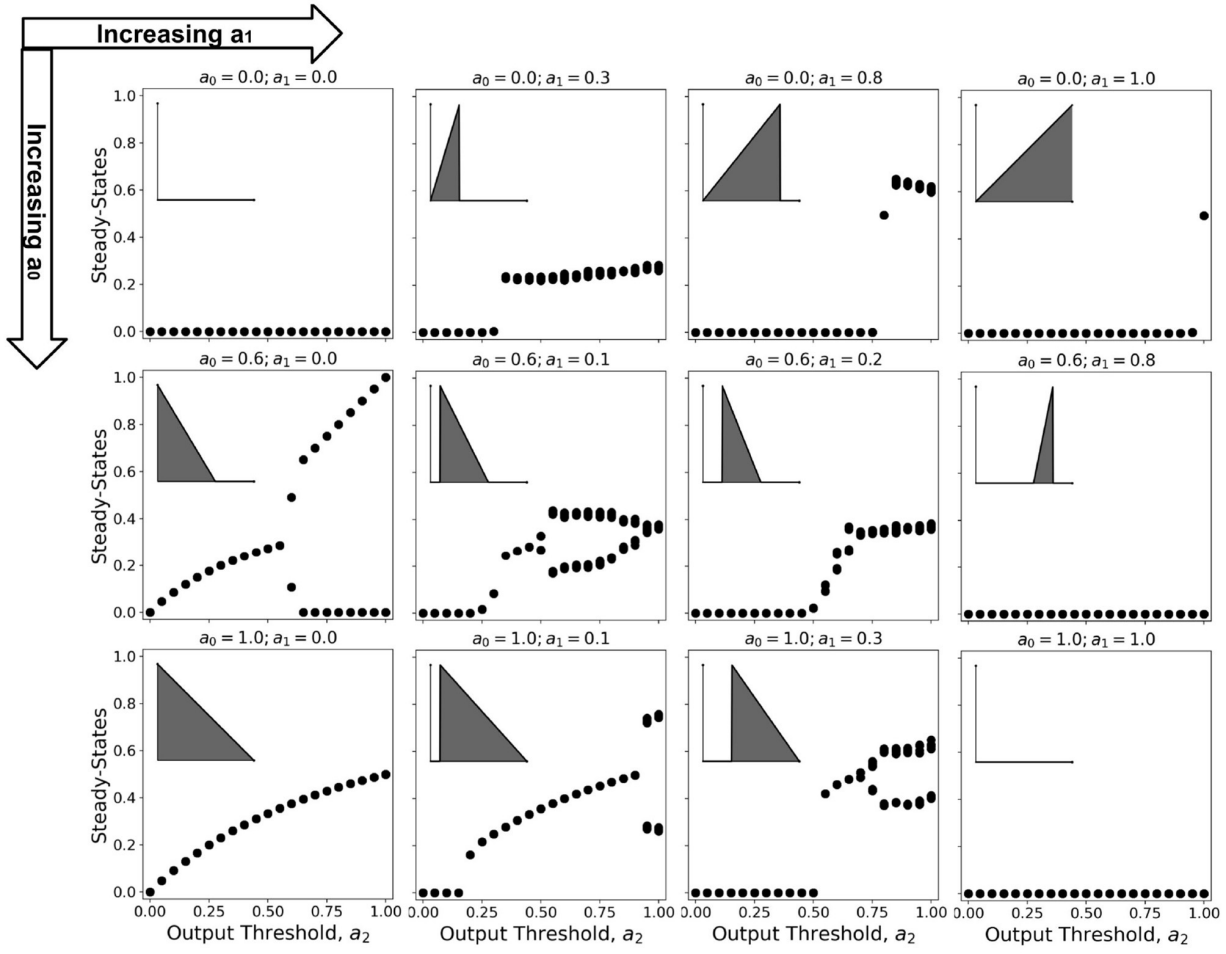
Representative bifurcation diagrams for the neuronal CA system. Each individual plot shows the steady-state values vs. increasing *a*_2_ with constant *a*_0_, *a*_1_ values. From left to right column shows the different steady-state trends with increasing *a*_1_ while from top row to bottom shows increasing *a*_0_. The inset shows the activation function for that parameter set given that *a*_2_ = 1.0, for reference.

Bifurcation diagrams show the dynamical trend of the system as we vary a parameter of interest Çelik Karaaslanl (2012). In this work, we chose to investigate the trend for varying *a*_2_-values, holding both *a*_0_ and *a*_1_ at various combinations of constant values. Since 100 timesteps is enough for the simulation to reach steady-state at any given parameter set Ramos and Bantang (2018, 2019c), we obtained the average neuronal patch activity 〈*a*〉 only for the last (10) timesteps. The resulting bifurcation diagrams are shown in Figure 11 (Ramos and Bantang, 2020).

**Figure 12:**
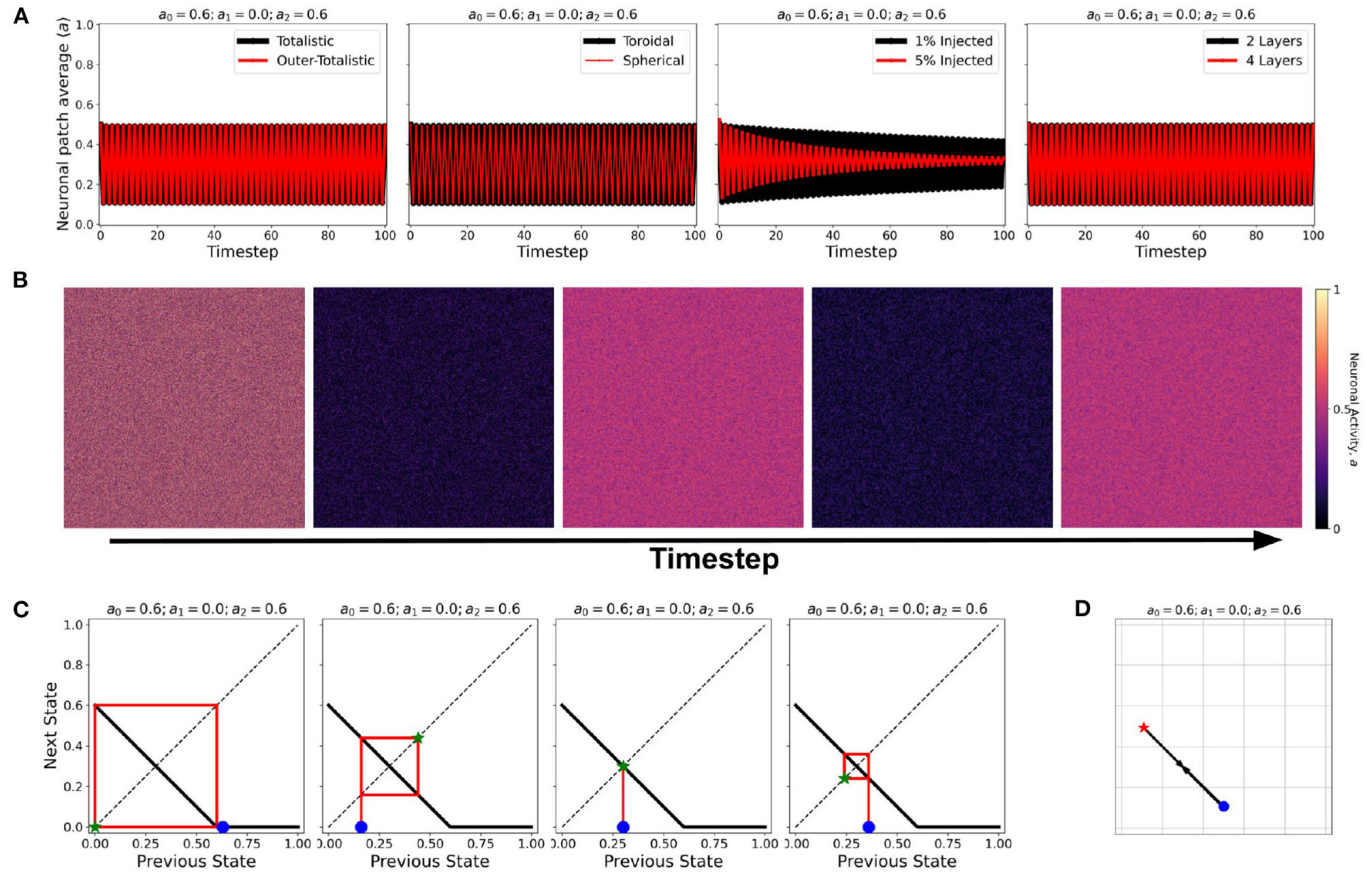
**(A)** Steady-state dynamics of Class 2 CA using the various neighborhood and boundary conditions aforementioned. **(B)** Snapshots showing spatiotemporal evolution of Class 2 CA. **(C)** Cobweb diagrams for each possible initial condition (marked by 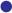) in a Class 2 CA with threshold values *a*_0_ = 0.6, *a*_1_ = 0.0, and *a*_2_ = 0.6. **(D)** Trajectory of the neuronal steady-state activity in state-space. The average state of the CA are marked at *t* = 0 (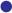) and at *t* = ∞ (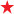) with the black arrows tracing the trajectory. It is notable that the neuronal CA cycles back and forth along the path of evolution.

At certain parameter sets, the neuronal CA exhibits period-doubling. This only happens when the activation function is negatively-sloped and strictly satisfies the conditions: *a*_1_ = 0 and *a*_0_>0. Only with this specific constraint will the overall neuron state oscillate as shown in the cobweb diagrams in Figure 12. An oscillating overall neuronal state indicates that a significant degree of synchronization happens in the majority fraction of the neurons. Epileptic neurons can be modeled by these negatively-sloped activation functions. This oscillatory behavior is unchanged by any neighborhood and boundary conditions, as shown in Figure 12. However, as we increase the fraction of neurons *c*_ext_ with input, the oscillation becomes underdamped.

## 7. Extending to Nonlinear Activation Function

**Figure 13:**
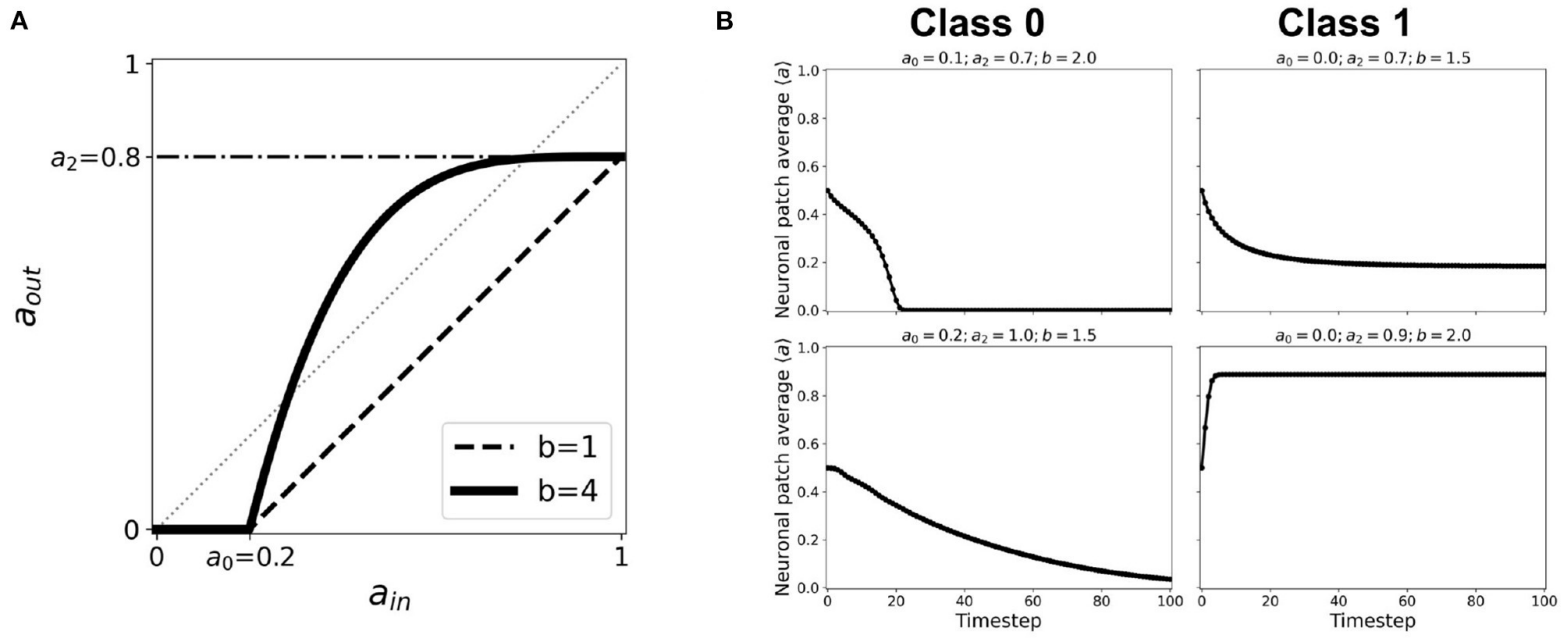
**(A)** Representative nonlinear activation function (*b* = 4) using the second-order approximation given by Equation 2 with thresholds *a*_0_ = 0.2, *a*_1_ = 1.0, *a*_2_ = 0.8. The black dashed line shows the same thresholds with nonlinearity parameter *b* = 1, which is equivalent to the case of first-order approximated activation function. **(B)** Representative steady-state dynamics for each class using nonlinear activation function (*b* > 1).

As discussed in section 1, one possible extension of the model is to consider a nonlinear activation function that provides a better approximation of the neuronal response (Hodgkin and Huxley, 1952; Gerstner et al., 2014; Pang and Bantang, 2015). The second-order approximation of the activation function is given by the equation:


(2) $$a_{\text{out}}=\;\{\begin{array}{ll} 0 & 0<a_{\text{in}}<a_{0}\\ a_{2}(1-{\left(1-\frac{a_{\text{in}}-a_{0}}{1-a_{0}}\right)}^{b}) & a_{\text{in}}\geq a_{0} \end{array}$$

where *a*_0_, *a*_2_ represents the same thresholds as in Equation 1, and *b* is the nonlinearity parameter. Here, *a*_1_ = 1. When *b* = 1, this function reverts to the first-order linear approximation. Figure 13A shows how the activation function changes when we increase *b*. An exhaustive testing of the nonlinear activation function has been done with varying input and output thresholds *a*_0_, *a*_2_∈[0, 1], and nonlinearity parameter *b*∈[0, 40] (Ramos and Bantang, 2021). The resulting dynamics are classified below. Representative steady-state dynamics for each class are shown in Figure 13B.

1. Class 0: Quiescent Steady-State: (a) Fast-decay; (b) Slow-decay
2. Class 1: Spiking Steady-State: (a) low activation probability; (b) high activation probability.

The conditions for phase transition are presented in a previous work (Ramos and Bantang, 2021). It is notable that the opposing extreme cases (*a*_2_ = 0.0 and *a*_0_ = 1.0) always belong to Class 0. Whenever 0 ≤ *b* < 1, the system also falls under Class 0 independent of the other parameters. Once we increase the nonlinearity such that *b*>1, especially for cases when the corresponding linear activation function lies completely in the region below the *a*_out_ = *a*_in_ line, the dynamical evolution transitions from Class 0 to Class 1. This transition is caused by the crossing of the activation function to the region above *a*_out_ = *a*_in_ line as shown in Figure 13A. Hence, using the cobweb analysis above, there are individual neurons that will contribute to an overall system spiking steady-state. We also found that, on one hand, the average steady-state activity transitions abruptly when the input threshold *a*_0_ is decreased. On the other hand, the transition is gradual when the output threshold *a*_2_ is increased, with the phase boundary approximated at *b*~1/*a*_2_. Furthermore, increasing the nonlinearity parameter b transitions the neuronal classification from Class 0 to Class 1.

It is notable that the opposing extreme cases (*a*_2_ = 0.0 and *a*_0_ = 1.0) always belong to Class 0. Whenever 0 ≤ *b* < 1, the system also falls under Class 0 independent of the other parameters. Once we increase the nonlinearity such that *b*>1, especially for cases when the corresponding linear activation function lies completely in the region below the *a*_out_ = *a*_in_ line, the dynamical evolution transitions from Class 0 to Class 1. This transition is caused by the crossing of the activation function to the region above *a*_out_ = *a*_in_ line as shown in Figure 13A. Hence, using the cobweb analysis above, there are individual neurons that will contribute to an overall system spiking steady-state.

## 8. Young and Aged Neuronal Systems

As an application of the proposed CA modeling paradigm, we obtained an empirical dataset of the single-cell response that shows the dynamical difference between young and aged neurons in response to input signals (Coskren et al., 2015). Data shows higher firing rates from the aged neurons. The dataset is normalized over the range of the input current (30−−330pA) used in the study. When the first-order approximation activation function *a*_out_ = *f*(*a*_in_) given by Equation 1 is used to fit the dataset, both young and aged neuronal system resulted to quiescent steady-state with young neuronal CA decaying faster than the aged ones (see Supplementary Figure 3). If injected *a*_ext_ = 1–5% of the neuronal population, the average steady-state is the same for both young and aged neuronal systems. This result contradicts the observations by Coskren et al. (2015).

**Figure 14:**
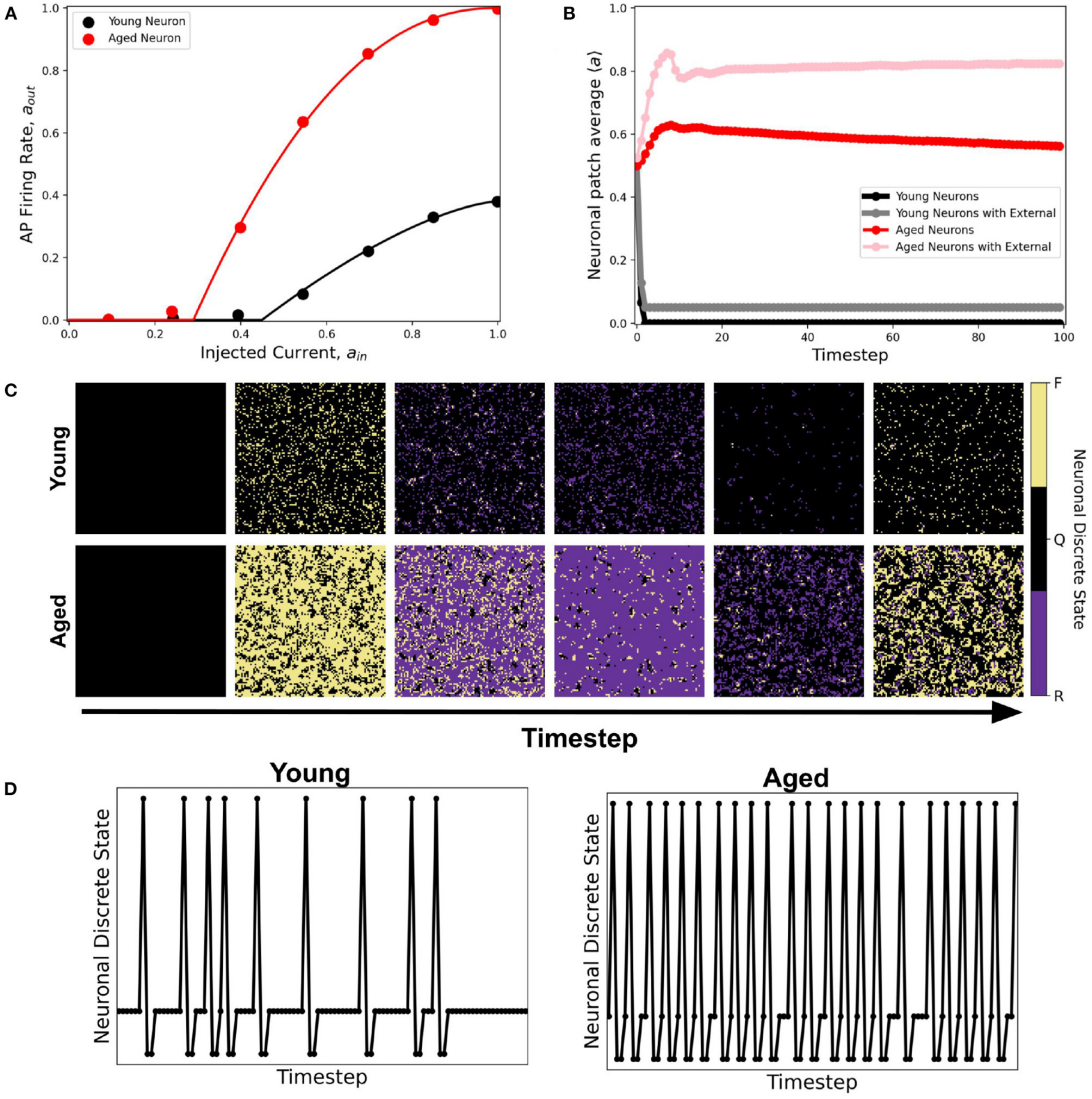
**(A)** Activation function of young (black) and aged (red) neuronal population derived from empirical data from rhesus monkey prefrontal cortex (Coskren et al., 2015). The dots are the dataset from the study, while the solid lines are the activation function fitted using Equation 2. The parameter thresholds are *a*_0_ = 0.45, *a*_2_ = 0.38, *b* = 1.5 for the young neuronal system, and *a*_0_ = 0.29, *a*_2_ = 1.0, *b* = 2.2 for the aged population. **(B)** Corresponding average steady-state dynamics of young and aged neuronal population using the CA model. **(C)** Actual response of young and aged neuronal patch obtained using the method described in Ramos and Bantang (2018) and Ramos and Bantang (2021). The color represents the discrete state the neuron is in: Q (Quiescent), F (Firing), R (Refractory). **(D)** Representative temporal response of a single neuron from the young and aged neuronal patch according to the discrete states in **(C)**.

A better fitting function to the dataset is the second-order approximation given by Equation 2. Figure 14A shows the resulting response curve. Using this response curve, we found a significant difference in the dynamics between young and aged neuronal systems (see Figure 14B). The aged neuronal population shows a spiking steady-state with a higher average neuronal response than the younger population. Injecting constant external input (*a*_ext_ = 1) randomly to 5% of the neuronal population amplifies the average steady-state for both young and aged neuronal systems but with different steady-state values. Hence, the aged neuronal system does not need a very high external input for it to be amplified, unlike the younger population. This result confirms the higher firing rate of aged neurons as well as the presence of spiking states for aged at lower input currents (Coskren et al., 2015). Figure 14C shows the actual response of young and aged neuronal patches obtained using the method described in Ramos and Bantang (2018) and Ramos and Bantang (2021). A sample discrete response of a single neuron from the young and aged neuronal patches is shown in Figure 14D.

## 9. Computational Complexity

The computational efficiency of using CA to model neuronal patch dynamics is quantified using the time it takes to finish a given simulation. With increasing neuronal population *N*, the time it takes to finish the simulation *T* is recorded as the average of three (3) runs or trials for each solver. In general, we find that the time *T* can be fitted with:

(3) $$\begin{array}{r} T=a{\left(N-b\right)}^{c} \end{array}$$

where *a*, *b* and *c* are the fitting parameters. From these parameters, *c* provides the relevant information for the computational complexity whereby *T*~*O*(*N*^*c*^) for large *N*-values.

**Figure 15:**
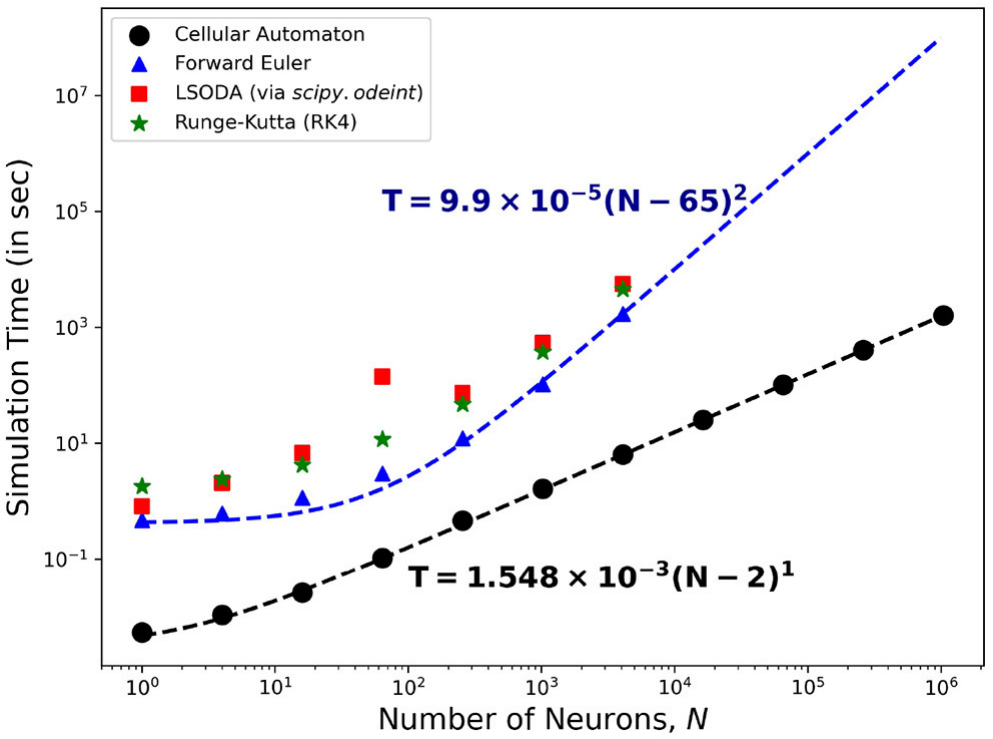
Time complexity of the neuronal model comparing different algorithms for solving the HH dynamics. The forward Euler method (marked 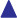) shows quadratic order in time (~*O*(*N*^2^)) while the CA model (marked •) presents a linear complexity time (~*O*(*N*)) indicating the efficiency of using the CA model in simulation of neuronal patch dynamics.

Figure 15 shows the comparison of the simulation time using different solvers to the HH ODEs and the CA modeling method presented here. On one hand, solving the ODEs of the HH neuronal network using the forward Euler method yields quadratic time complexity (*T*~*O*(*N*^2^)). Using more accurate solver variants such as Runge-Kutta order-4 (RK4) and Livermore Solver for Ordinary Differential Equations(LSODA) increases the overall magnitude of *T* yet returns consistent complexity *c*≈2. On the other hand, our CA model presents a linear time complexity (*T*~*O*(*N*)) indicating a much faster computational time than simulating interconnected HH neurons, especially for much larger system size *N*.

Figure 15 also shows that the *T*-values for HH neuronal population sizes beyond *N* = 4, 096 is absent. Running simulations for larger sizes causes our current computational machines to exceed their memory capacity. The use of our CA model shows significant advantage in simulating up to millions of neurons (more than 10^3^ times the other reported approach) before this memory problem happens. The algorithm of the CA model can be more straightforwardly parallelized and GPU-implemented to amplify the neuronal population without increasing the simulation time.

## 10. Conclusions

In this work, we proposed a cellular automata (CA) model as an efficient way of modeling large numbers of neurons that can reduce both computational time and memory requirements in simulation. We implemented neuronal dynamics on a neuronal CA patch of lattice size 1, 024 × 1, 024 using a first-order linear approximation of the resulting activation function of the HH model. The system dynamics is characterized according to the three parameters of the resulting activation function. The steady-state dynamics are investigated for different lattice configuration (2D and quasi-3D), boundary conditions (toroidal and spherical), layering (one- or two-layered), and Moore neighborhood type (totalistic and outer-totalistic). Cases wherein a fraction (1% and 5%) of neurons have constant activation input (*a*_in_) are also explored. We observed the following CA classification:

1. Class 0: Quiescent Steady-State: (a) Fast-decay; (b) Slow-decay
2. Class 1: Spiking Steady-State:(a) With random patterns; (b) With exploding patterns
3. Class 2: Oscillatory Steady-State.

Numerical experiments of CA neuronal systems are shown to conform to this classification. While our analysis of the cobweb diagrams show that individual neuron states will eventually reduce to quiescent state, spiking steady-state can still emerge for a collection of interconnected neurons. Collective oscillatory behavior (Class 2) of the overall neuronal state is observed for the system with significant synchronization among neurons.

The proposed CA model is applied to analyze the resulting dynamical class of young and aged patches of neurons. The response function of individual young and aged neuronal cells are obtained from empirical data and are fitted to a second-order approximation for better semblance. The CA model for aged patch shows dynamics with higher average neuronal steady-state and therefore more robust spiking behavior compared to the younger population. This result conforms to the higher action potential firing rates of aged neurons from the empirical data. On one hand, the average neuronal steady-state is amplified for the aged population when injected with a small external input. On the other hand, the younger population needs higher external input to observe significant amplification of the average neuronal steady-state. This result conforms to the presence of spiking activity in aged neurons stimulated with lower external current. Whether artificially generated spatiotemporal patterns of neuronal patch activity in this work correspond to the activity of actual neuronal systems remains to be determined.

The cellular automata model presented here can easily be extended to model more realistic neuronal systems such as brain patches or even the whole brain. Individual neuronal response data can also be used to improve the choice of the CA activation function *a*_out_ = *f*(*a*_in_). The activation function can be modified into similar input-output mapping in frequency domain or voltage-current domain, and can be used as the rule for our CA model. Our computational modeling framework can be utilized for large scale simulation of different neuronal conditions such as Parkinson's disease (Bevan et al., 2002; Kang and Lowery, 2014), Alzheimer's disease, and chronic traumatic encephalopathy (Gabrieli et al., 2020; Wickramaratne et al., 2020).

We presented here that an adult brain shows an increase of neuronal response, even in the presence of constant external input. However, it remains a challenge to understand in which particular biological aspect these changes correspond to. In future studies, we recommend investigating dynamical systems of interconnected neurons, both young and aged, in the following aspects: 1) input-output mapping; 2) spatiotemporal distribution; and 3) connectivity architecture. Learning about the dynamics of these systems would help medical practitioners to detect early signs of ailments or disorders stemming from the aging process and help identify appropriate medicinal (chemical, radiation), behavioral (lifestyle, dietary) and/or surgical intervention.

## Data Availability Statement

The original contributions presented in the study are included in the article/Supplementary Material, further inquiries can be directed to the corresponding author.

## Author Contributions

RR did the simulation and wrote the manuscript draft. All authors conceived the research problem and analyzed the results, and edited the final manuscript.

## Conflict of Interest

The authors declare that the research was conducted in the absence of any commercial or financial relationships that could be construed as a potential conflict of interest.

## Publisher's Note

All claims expressed in this article are solely those of the authors and do not necessarily represent those of their affiliated organizations, or those of the publisher, the editors and the reviewers. Any product that may be evaluated in this article, or claim that may be made by its manufacturer, is not guaranteed or endorsed by the publisher.
