# Supplementary material for: Young and Aged Neuronal Tissue Dynamics With a Simplified Neuronal Patch Cellular Automata Model
Source: Front Neuroinform. 2022 Jan 7;15:763560. doi: 10.3389/fninf.2021.763560 (PMC8777299; doi:10.3389/fninf.2021.763560)
Supplement: Supplementary file 1 [file Presentation_1.PDF]

Supplementary Material

1 SUPPLEMENTARY FIGURES

1.1 Toroidal CA vs Spherical CA

Figure S1 compares the average neuronal state between toroidal CA and spherical CA. The steady-state remains unchanged indicating that the proposed neuronal classification scheme holds for both CA lattice configurations.

1.2 Two-Layered Lattice Dynamics

The average neuronal state for each layer in a two-layered lattice CA is shown in Figure S2. For each class, the neuronal dynamics remains unchanged for all layers and is also the same with the average of the whole layered lattice system.

1.3 Young vs Aged Neuronal Population with Linear Activation Function

Figure S3A shows the activation function of young and aged neuronal population using an empirical dataset of rhesus monkey prefrontal cortex (Coskren et al., 2015). The dataset is fitted using the following equation:

$$a_{\text{out}} = \begin{cases} 0 & a_{\text{in}} < a_0 \\ \frac{a_2(a_{\text{in}} - a_0)}{a_1 - a_0} & a_{\text{in}} \in [a_0, a_1] \\ 0 & a_{\text{in}} > a_1 \end{cases} \tag{S1}$$

The resulting dynamics (see Figure S3B) shows similar steady-state behavior for both young and aged population. When subjected with external input, both young and aged population amplifies to the same average steady-state value. These results is contrary to what was observed by Coskren et al. (2015). Hence, the activation function is modified into the second-order approximation for better semblance.

2 SUPPLEMENTARY VIDEO ANIMATIONS

The supplementary movie files (mp4 format) show the evolution of 1024×1024 neuronal lattice with an outer-totalistic toroidal configuration for each classes.

SuppFigure4. Spatiotemporal Evolution of Class 0a CA

SuppFigure5. Spatiotemporal Evolution of Class 0b CA

SuppFigure6. Spatiotemporal Evolution of Class 1a CA

SuppFigure7. Spatiotemporal Evolution of Class 1b CA

SuppFigure8. Spatiotemporal Evolution of Class 2 CA

REFERENCES

Coskren PJ, Luebke JI, Kabaso D, Wearne SL, Yadav A, Rumbell T, et al. Functional consequences of age-related morphologic changes to pyramidal neurons of the rhesus monkey prefrontal cortex. *Journal of computational neuroscience* **38** (2015) 263–283. doi:10.1007/s10827-014-0541-5.

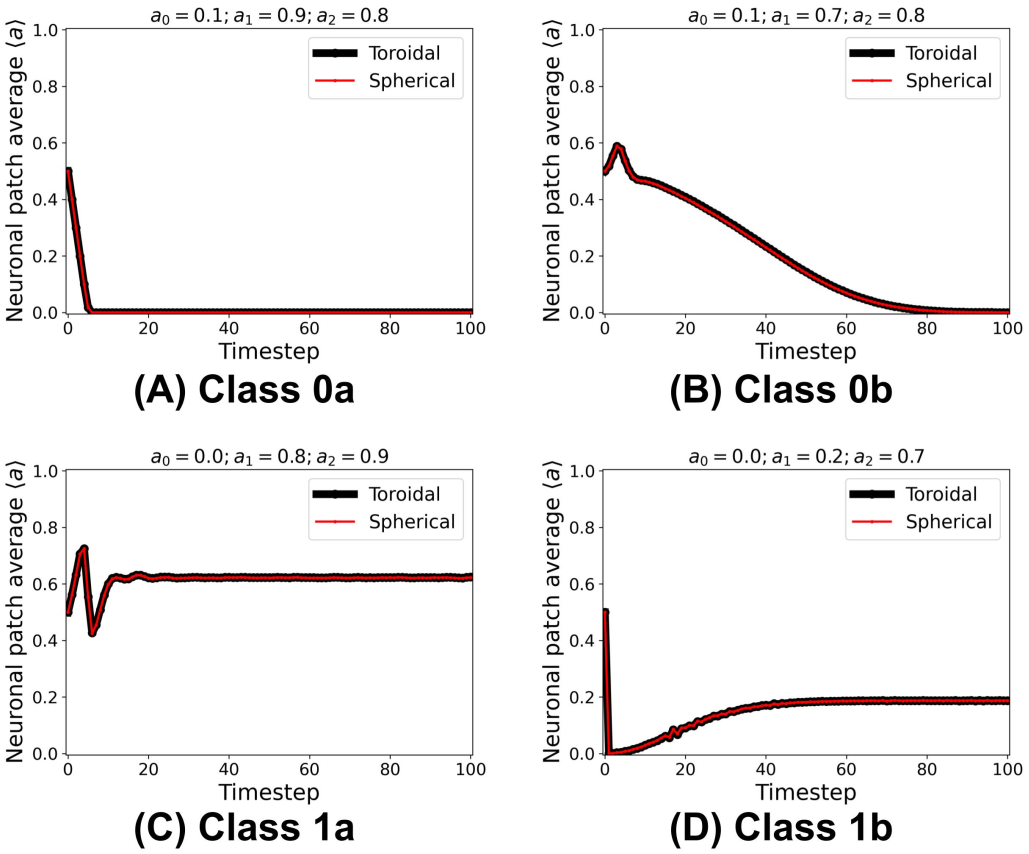

Figure S1. Steady-state dynamics of each class in a toroidal (black line) and spherical (red line) lattice configuration. The dynamical trend remains the same for each class.

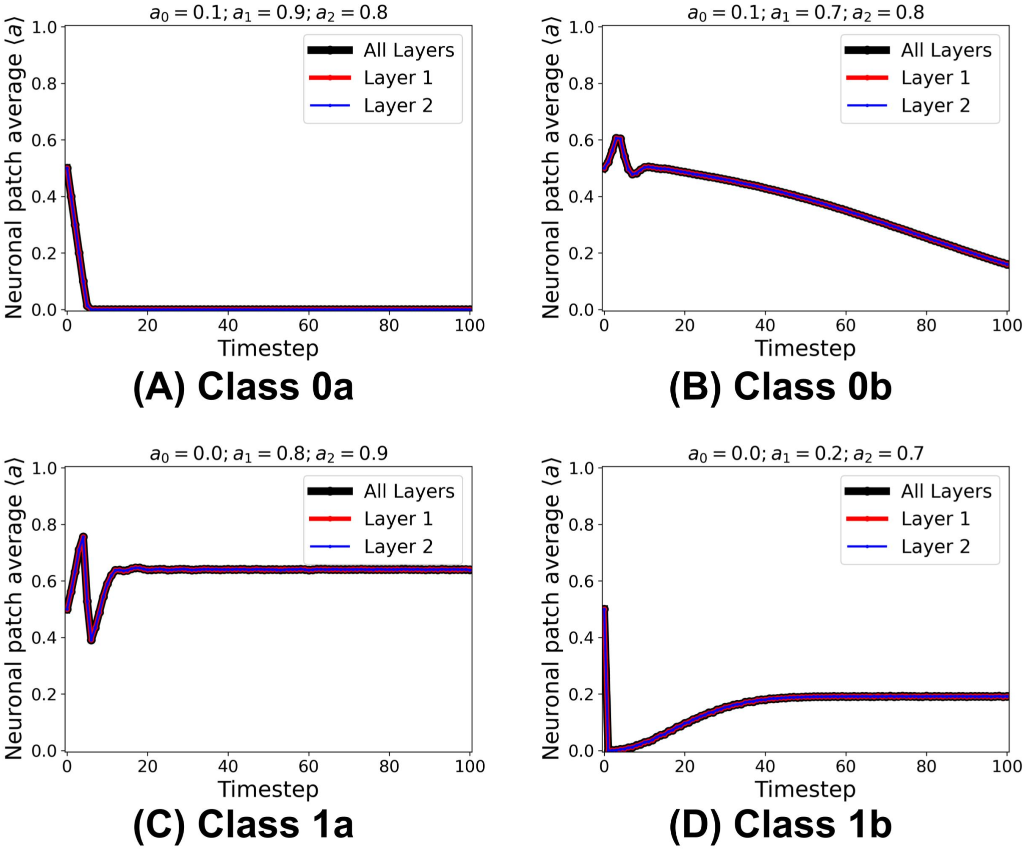

Figure S2. Steady-state dynamics of each class in a two-layered toroidal lattice. The average neuronal activity is the same across each layer and has similar dynamics with the average activity of the whole CA system.

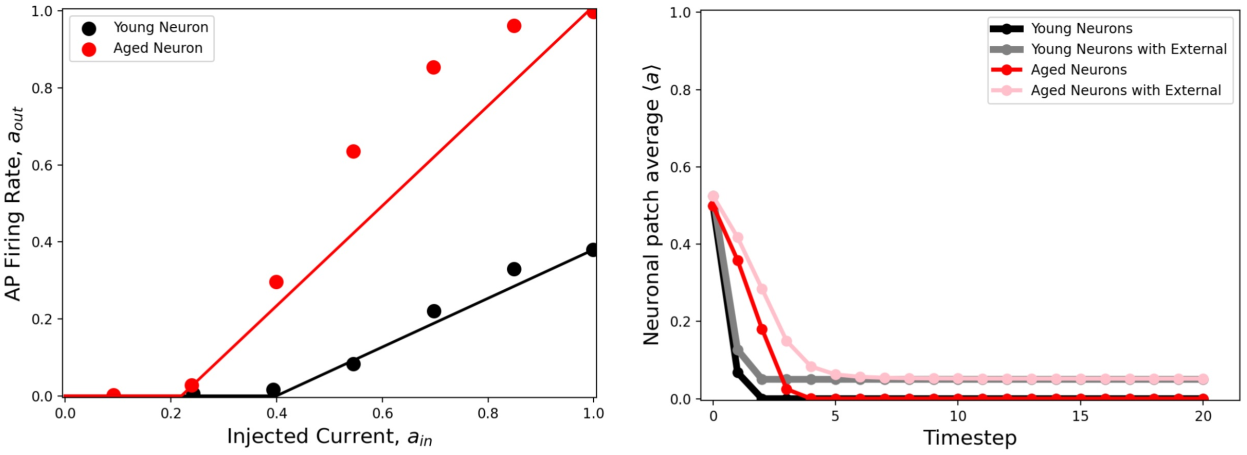

(A) Normalized activation function derived from empirical data (Coskren et al.)

(B) Steady-state dynamics of the CA system of young and aged neuronal populations

Figure S3. **(A)** Activation function of young (black) and aged (red) neuronal population derived from empirical data from rhesus monkey prefrontal cortex (Coskren et al., 2015). The dots are the dataset from the study, while the solid lines are the activation function fitted using the linear activation function Equation S1. The parameter thresholds are $a_0 = 0.4,\ a_1 = 1.0,\ a_2 = 0.38$ for the young neuronal system, and $a_0 = 0.22,\ a_1 = 1.0,\ a_2 = 1.0$ for the aged population. **(B)** Corresponding average steady-state dynamics of young and aged neuronal population using the CA model.
